# Evolving Role of Catheter Ablation for Atrial Fibrillation: Early and Effective Rhythm Control

**DOI:** 10.3390/jcm11226871

**Published:** 2022-11-21

**Authors:** Shaojie Chen, Yuehui Yin, Zhiyu Ling, Christian Meyer, Helmut Pürerfellner, Martin Martinek, Márcio Galindo Kiuchi, Piotr Futyma, Lin Zhu, Alexandra Schratter, Jiazhi Wang, Willem-Jan Acou, Philipp Sommer, Feifan Ouyang, Shaowen Liu, Julian K. R. Chun, Boris Schmidt

**Affiliations:** 1 Cardioangiologisches Centrum Bethanien (CCB), Kardiologie, Medizinische Klinik III, Agaplesion Markus Krankenhaus, Akademisches Lehrkrankenhaus der Goethe-Universität Frankfurt am Main, 60431 Frankfurt am Main, Germany; 2 Die Sektion Medizin, Universität zu Lübeck, 23538 Lübeck, Germany; 3 Department of Cardiology, The Second Affiliated Hospital of Chongqing Medical University, Chongqing 400016, China; 4 Department of Cardiology, cNEP, Cardiac Neuro- & Electrophysiology Research Group, University Heart & Vascular Center Hamburg, University Hospital Hamburg-Eppendorf, 20246 Hamburg, Germany; 5 DZHK (German Center for Cardiovascular Research), Partner Site Hamburg/Kiel/Lübeck, 20246 Hamburg, Germany; 6 Department of Cardiology, Evangelical Hospital Düsseldorf, 40225 Düsseldorf, Germany; 7 Heinrich Heine University Hospital Düsseldorf, 40225 Düsseldorf, Germany; 8 Department für Kardiologie und Elektrophysiologie, Akademisches Lehrkrankenhaus, Ordensklinikum Linz Elisabethinen, 4020 Linz, Austria; 9 School of Medicine-Royal Perth Hospital Unit, University of Western Australia, Perth, WA 6000, Australia; 10 St. Joseph’s Heart Rhythm Center, Medical College, University of Rzeszów, 35-959 Rzeszów, Poland; 11 Medizinisch-Geriatrische Klinik, Agaplesion Markus Krankenhaus, Akademisches Lehrkrankenhaus der Goethe-Universität Frankfurt am Main, 60431 Frankfurt am Main, Germany; 12 Abteilung für Kardiologie, Klinik Floridsdorf Wien, 1210 Vienna, Austria; 13 Intensivmedizin, Charité—Universitätsmedizin Berlin, 12203 Berlin, Germany; 14 Department of Cardiology, AZ Delta, 8800 Roeselare, Belgium; 15 Klinik für Elektrophysiologie/Rhythmologie, Herz- und Diabeteszentrum Nordrhein-Westfalen, Universitätsklinik der Ruhr-Universität Bochum, 32545 Bad Oeynhausen, Germany; 16 Klinik und Poliklinik für Kardiologie, Universitäres Herz und Gefäßzentrum, Universitätsklinikum Hamburg-Eppendorf (UKE), 20246 Hamburg, Germany; 17 Department of Cardiology, Shanghai General Hospital, Shanghai Jiao Tong University School of Medicine, Shanghai 200080, China

**Keywords:** atrial fibrillation, ablation, rhythm control

## Abstract

Catheter Ablation (CA) is an effective therapeutic option in treating atrial fibrillation (AF). Importantly, recent data show that CA as a rhythm control strategy not only significantly reduces AF burden, but also substantially improves clinical hard endpoints. Since AF is a progressive disease, the time of Diagnosis-to-Intervention appears crucial. Recent evidence shows that earlier rhythm control is associated with a lower risk of adverse cardiovascular outcomes in patients with early AF. Particularly, CA as an initial first line rhythm control strategy is associated with significant reduction of arrhythmia recurrence and rehospitalization in patients with paroxysmal AF. CA is shown to significantly lower the risk of progression from paroxysmal AF to persistent AF. When treating persistent AF, the overall clinical success after ablation remains unsatisfactory, however the ablation outcome in patients with “early” persistent AF appears better than those with “late” persistent AF. “Adjunctive” ablation on top of pulmonary vein isolation (PVI), e.g., ablation of atrial low voltage area, left atrial posterior wall, vein of Marshall, left atrial appendage, etc., may further reduce arrhythmia recurrence in selected patient group. New ablation concepts or new ablation technologies have been developing to optimize therapeutic effects or safety profile and may ultimately improve the clinical outcome.

## 1. Introduction

Atrial fibrillation (AF) is a common cause of ischemic stroke, cardiac dysfunction, and cardiovascular death, leading to poor health-related quality of life (QoL) and increased hospitalization [1]. The overall prevalence of AF is around 1% to 2%. The prevalence of AF has been increased 3-fold over the last 50 years, and it is expected to double in the next decades [1]. As the most common cardiac arrhythmia, AF has a dramatic impact on the burden of healthcare worldwide.

Rate and rhythm control combined with prevention of ischemic stroke/embolism are the fundamental treatment strategies for AF [1,2]. Catheter ablation (CA) as a rhythm control strategy is based on the knowledge that the main focal triggers for paroxysmal AF locate within the pulmonary vein (PV) muscular sleeves. CA has been shown as an effective therapeutic option in treating AF, and pulmonary vein isolation (PVI), using radiofrequency (RF), cryoballoon (CB), or other validated energy sources, forms the cornerstone ablation approach [1].

International guidelines primarily recommend CA for symptomatic paroxysmal/persistent AF patients who are refractory or intolerant to antiarrhythmic drugs (AADs) therapy [1,3]. Along with continuous advancement in ablation technology and increasingly accumulated clinical evidence, the role of CA has been evolving.

## 2. CA as First Line Therapy in Treating Paroxysmal AF

### 2.1. The Concept of Diagnosis-to-Intervention Time

**Figure 1:**
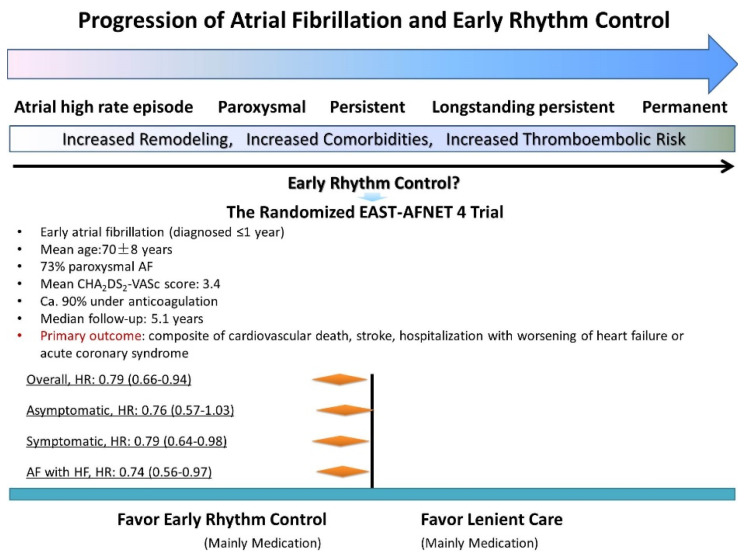
Progression of Atrial Fibrillation and Early Rhythm Control.

AF is a progressive disease. Current consensus is that, the majority of the AF patients first develop paroxysmal AF where ectopic firings in the pulmonary vein act as the main mechanism responsible for AF initiation. Paroxysmal AF can then progress to persistent or even long-standing AF if left untreated due to electrical or structural remolding. Therefore, the Time of “Diagnosis-to-(effective)-Intervention” appears crucial (Figure 1).

### 2.2. Early AADs Rhythm Control vs. Lenient Rate Control

The most recent large EAST-AFNET-4 trial demonstrated that “early” rhythm control strategy was associated with a significantly lower risk of adverse cardiovascular outcomes than usual care [4]. Notably, the EAST-AFNET-4 trial represented an early AF patient group with mean age of 70 ± 8 years old, 73% paroxysmal AF, mean CHA_2_DS_2_-VASc score of 3.4, and ca. 90% rate of anticoagulation. In the early rhythm control group 87% of the patients received AADs as initial therapy whereas only 8% of the patients received CA. Nonetheless, the EAST-AFNET-4 trial underscored the importance of early rhythm control rather than lenient rate control [4] (Figure 1).

### 2.3. Early Ablation Rhythm Control vs. AADs Rhythm Control

**Figure 2:**
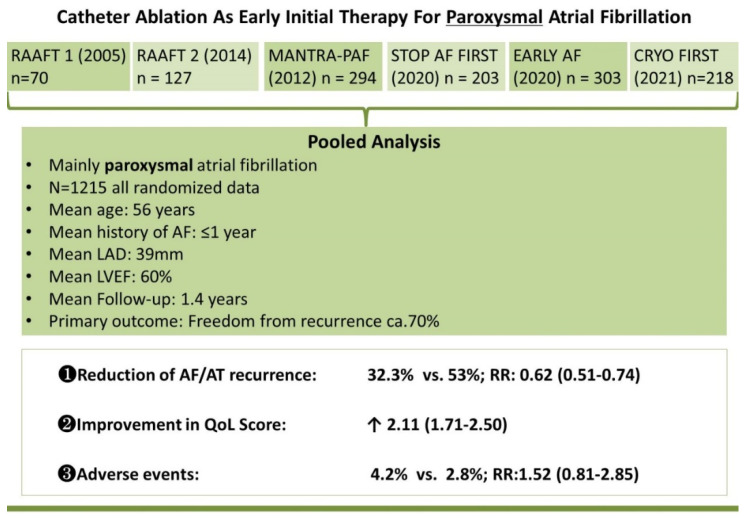
Catheter Ablation as Early Initial Therapy for Paroxysmal Atrial Fibrillation.

CA as the early initial therapy in treating AF has been a debate. To date, six randomized controlled trials (RCT) investigated the role of CA as first line therapy for AF [5,6,7,8,9,10] (Figure 2). Of them, three previous RCTs employed radiofrequency (RF) technology [8,9,10], whereas three recent RCTs employed cryoballoon (CB) technology [5,6,7]. A recent meta-analysis combined the 6 RCTs, the baseline characteristics of the study population from those RCTs were: mainly paroxysmal AF, mean age 56 years, mean history of AF 1 year, mean left atrial diameter (LAD) 39 mm, mean left ventricular ejection fraction (LVEF) 60%. By combining more than 1200 patients, the results showed that, CA compared with AADs was associated with significant reductions in recurrent atrial arrhythmia (32.3% vs. 53%; risk ratio (RR): 0.62; 95%CI: 0.51–0.74; *p* < 0.001) and rehospitalization, (5.6% vs. 18.7%; RR: 0.32; 95%CI: 0.19–0.53; *p* < 0.001) with no significant difference in serious adverse events between the CA and the AADs group (4.2% vs. 2.8%; RR: 1.52; 95%CI: 0.81–2.85; *p* = 0.19) [11]. Pooled-analysis from our group showed consistent results favoring CA in either 1 year or 2 years follow-up, and significantly greater improvement in QoL [12] (Figure 2). We also found that PVI using CB or RF had similar efficacy in maintaining sinus rhythm and overall similar rate of procedure related adverse events, and CB appeared to be associated with less procedural time and lower rate of cardiac tamponade [12]. These results seemed to be consistent with the previous FIRE AND ICE trial and the recent CIRCA-DOSE trial [13,14].

## 3. Effect of CA on Progression of AF

**Figure 3:**
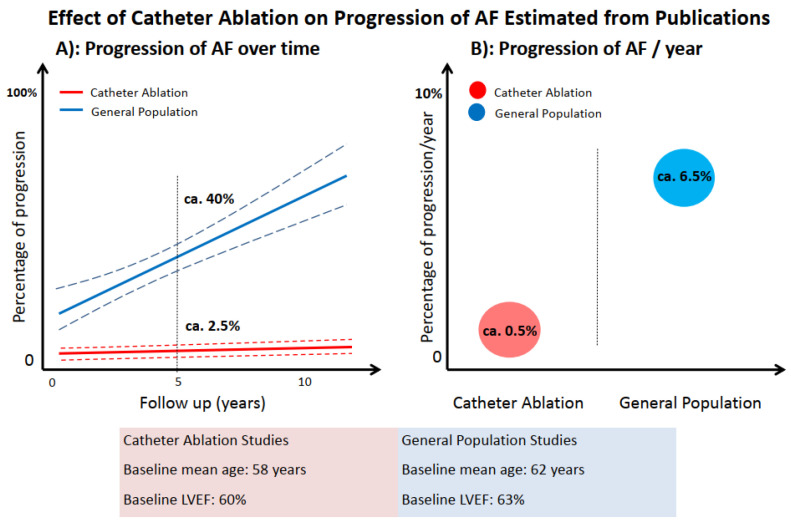
Effect of Catheter Ablation on Progression of AF Estimated from Publications.

Would CA prevent the progression of AF? A previous meta-analysis included twenty-one studies of 12,967 patients (mean age: 62.3 ± 2.4 years, mean LVEF: 63.2 ± 2.5%) with paroxysmal AF under medical therapy without CA, and the results revealed that the percentage of AF progression at 1 year ranged from 10% to 20%, and studies that had a longer follow-up detected a higher percentage of progression (from 50% to 77% after 12 years) [15], whereas meta-analysis of patients with paroxysmal AF undergoing CA (N = 2767, mean age: 58.2 ± 3.2 years, mean LVEF: 60.4 ± 2.9%) revealed significantly lower percentage of progression (from 2.4% to 2.7% at 5 years’ follow-up) with estimation of 0.5% per year [15]. These observations indicated CA may prevent the progression of AF (Figure 3). The recent randomized ATTEST trial also demonstrated that CA can significantly delay the AF progression (0.8% per year) as compared with AADs (5.8% per year) [16].

Moreover, previous pooled-analysis of 4950 patients demonstrated that Diagnosis-to-Ablation time ≤ 1 year was associated with a significantly lower relative risk of AF recurrence compared with that > 1 year (RR: 0.73, 95% CI, 0.65–0.82), suggesting that a shorter Diagnosis-to-Ablation time may be associated with a substantially increased success rate of CA [17].

## 4. CA in Treating Persistent AF (Figure 4)

Persistent AF represents the advanced stage during the progression of AF. “AF begets AF” is recognized as a main mechanism for the persistence of AF: a complex situation involving triggers and substrate (i.e., structural, electrical, and autonomic remodeling). Previous meta-analysis of RCTs including 809 persistent AF patients (mean age 60 years, mean LAD 46 mm) has already shown that PVI based CA is superior to AADs in preventing recurrence of atrial tachyarrhythmia among patients with persistent AF [18]. Nonetheless, the clinical success after ablation of persistent AF remains unsatisfactory and the optimal ablation strategy for persistent AF is to be determined.

**Figure 4:**
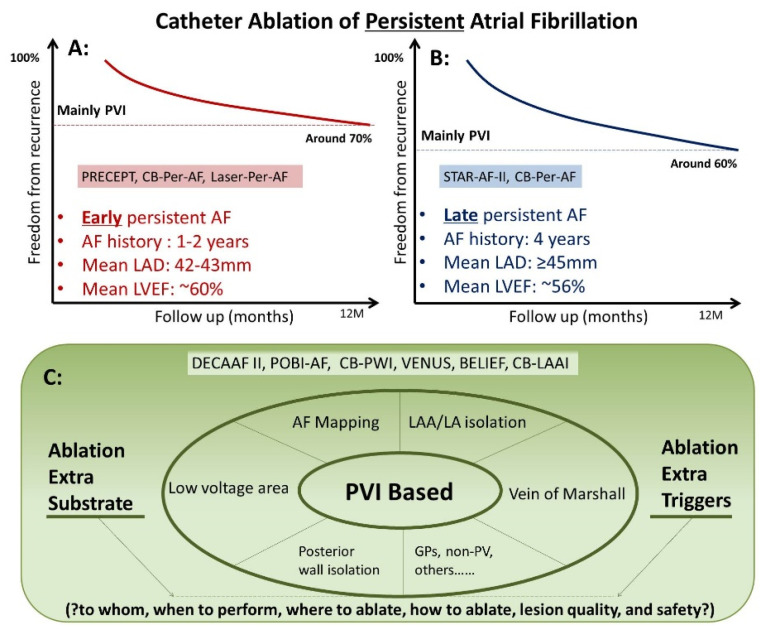
Catheter Ablation of Persistent Atrial Fibrillation. (**A**) Catheter ablation in “early” persistent AF. (**B**) Catheter ablation in “late” persistent AF. (**C**) Catheter ablation in non-PV sites.

### 4.1. CA in Treating Persistent AF and Longstanding Persistent AF

The recent PRECEPT trial employed contact force-sensing RF catheter guided by 3-D mapping system and lesion formation surrogates for PVI based ablation of persistent AF (N = 333, mean age 65 years, history of AF 1.3 years, mean LAD 42 mm, mean LVEF 56%). Linear ablation was only performed in cases with documented macro-re-entry atrial tachycardia (AT) or typical atrial flutter (AFL). Using standard electrocardiogram (ECG)/Holter monitoring, the PRECEPT study demonstrated a single procedure success rate around 70% at one year [19]. Randomized trials using different technologies for PVI in treating relatively healthy patients with persistent AF (mean age 66 years, history of AF 2 years, mean LAD 43 mm, mean LVEF 61%) reported similar ablation success rate around 70% at one year [20,21].

The randomized STAR AF II trial compared PVI vs. PVI plus (linear ablation vs. complex fractionated electrograms ablation) among patients with “late” persistent AF (N = 589, mean age 60 years, history of AF 4 years, mean LAD 45 mm, mean LVEF 56%). Using standard ECG/Holter monitoring, the STAR AF II study showed that 60% of the patients in the PVI group were free from recurrent AF during one year follow-up, and the study found no further reduction in AF recurrence when linear ablation or ablation of complex fractionated electrograms was performed in addition to PVI [22].

The ablation success rate appeared to be notably higher among patients with early persistent AF than those with late persistent AF, and this seemed to be consistent with the results from other recent studies [23,24,25,26].

### 4.2. Adjunctive Ablation Strategies beyond PVI in Treating Persistent AF

#### 4.2.1. Ablation of Atrial Low Voltage Area

Structural remodeling characterized by atrial fibrosis and enlargement is often accompanied during the progression of AF, and it is known that dilated atrium is an independent risk factor of recurrent arrhythmia after rhythm control. The previous DECAAF Study demonstrated that AF patients who had early-stage atrial fibrosis carried relatively lower risk of recurrent atrial tachyarrhythmia (15.3%, 95% CI: 7.6–29.6%) as compared to those who had late-stage atrial fibrosis 51.1% (95% CI: 32.8–72.2%) [27]. Substrate formation has been recognized as an important mechanism for arrhythmia. Elimination of the arrhythmogenic ventricular substrate has been shown to suppress ventricular arrhythmias in patients with ischemic cardiomyopathy; however, whether or not additional ablation of the fibrotic atrial substrate further improves the success of CA in AF remains controversial.

The most recent DECAAF II trial tested the hypothesis that magnetic resonance imaging (MRI) guided atrial fibrosis area ablation in addition to PVI is superior to PVI alone in improving ablation success in patients with persistent AF [28]. A total of 843 patients (mean age 62.1 years) were randomized, patients were categorized in I (mild)-IV (severe) stages according to atrial fibrosis level. The study found that, consistent with the results of the initial DECAAF study, the extension of baseline atrial fibrosis level was correlated with atrial tachyarrhythmia recurrence after CA. Results after CA based on intention-to-treat (ITT) analysis showed no significant difference in atrial tachyarrhythmia (ATa) recurrence between groups in the overall study population during one year follow-up, Interestingly, per protocol analysis showed significantly less ATa recurrence of substrate ablation in patients with stage I or II (<20%) atrial fibrosis, whereas there was no benefit of fibrosis ablation on ATa recurrence in patients with stage III or IV atrial fibrosis [28].

#### 4.2.2. Left Atrial Posterior Wall Isolation

The Left atrial posterior wall connecting the septal and lateral PVs has been identified as an arrhythmogenic region contributing to the initiation and maintenance of AF. Electrical isolation the left atrial posterior wall (PWI) has been shown to improve the ablation outcome.

The recent POBI-AF trial randomized 217 patients with persistent AF (73.3% longstanding persistent AF) to ablation with PVI alone or PVI + PWI. The freedom from ATa recurrence showed no significant difference between the PVI + PWI vs. PVI group (55.9% vs. 50.5%) after a mean follow-up of 16 months [29].

Cryoballoon (CB) has been established as a powerful tool for PVI and may create a more homogeneous and durable lesion [30]. A recent RCT investigated the effects of concomitant PVI + PWI using CB. One hundred and ten persistent AF (35% LS AF, mean LAD: 44 mm, mean LVEF 60%) were randomized into PVI or PVI + PWI. RF touch up was required in 7.3% patients to complete PVI and in 45% patients to complete PWI. One year follow-up demonstrated significantly higher freedom from ATa recurrence in the PVI + PWI group vs. PVI (74.5% vs. 54.5%) without compromising safety [31].

Recent large sized meta-analysis included 26 studies with 3287 patients with AF (age 61.7 ± 10.8 years) [32]. Procedural success to achieve PWI was 92.8%. The mean follow-up was 15.2 ± 8.4 months. For patients with paroxysmal AF, adjunctive PWI did not reduce the recurrence of all atrial arrhythmias or AF; whereas for patients with persistent AF, adjunctive PWI was associated with substantially lower recurrence of all atrial arrhythmias and AF, this finding remaining consistent when randomized data were included. Adjunctive PWI using either radiofrequency or a cryoballoon reduced AF recurrence, and primarily using cryoballoon seemed to be associated with lower recurrence rate of atrial tachycardias and/or atrial flutter. The incidence of procedural adverse events between PVI + PWI (3.2%) and PVI (2.8%) groups was low and similar. This meta-analysis indicated that patients with persistent AF appear to benefit from adjunctive PWI, and the ablation technology and/or approach may affect the clinical outcome of PWI [32].

#### 4.2.3. Ablation Vein of Marshall

The vein (ligament) of Marshall (VOM) contains innervation and electrical triggers to initiate AF. VOM ethanol infusion has been shown as a feasible treatment for atrial tachyarrhythmia by facilitating the block of mitral isthmus [33]. Repeat procedure data also showed that VOM ethanol infusion was associated with greater lesion durability and higher rate of sustained mitral isthmus block as compared with radiofrequency catheter ablation [34]. The recent VENUS trial randomized 343 persistent AF patients (53% LS AF, mean LAD 46, LVEF 53%) to CA or CA + VOM Ethanol Infusion. VOM Ethanol Infusion was successful in 84% of patients. One year follow-up showed that CA + VOM ethanol infusion was associated with significantly higher freedom from ATa recurrence as compared with CA (49.2% vs. 38%) without compromising the safety [35]. The post hoc analysis showed that adjunctive VOM ethanol infusion improved the ablation outcomes (59% vs. 39.1%) only when associated with effective mitral isthmus block [36].

#### 4.2.4. Left Atrial Appendage Isolation/Ablation

Left atrial appendage (LAA) has been recognized as a trigger source of AF/AT, and left atrial appendage isolation (LAAI) may suppress AF/AT recurrence. The previous BELIEF Trial randomized 173 long-standing persistent AF into LAAI plus standard ablation or standard ablation [37]. In this study, standard ablation was defined as PVI, PWI, left atrial (LA) lines, and non-PV triggers ablation. The mean age was 64 years, mean LAD 48 mm, mean LVEF 54%. During a 12-month follow-up, adjunctive LAAI was associated with significantly higher freedom from ATa as compared with standard ablation (56% vs. 28%). Notably, trigger from the LAA was identified in about one third of the patients during isoproterenol challenge [37]. 

A recent meta-analysis included nine studies with a total of 2336 patients. The majority of the patients were persistent AF or long-standing persistent AF, with a mean age of 65 years, mean LAD 45 mm, mean LVEF 56%. During a mean follow-up of 41 months, patients who underwent LAAI had significantly higher freedom from ATa recurrence than patients who underwent standard ablation (69.3% vs. 46.4%), and there was no significant difference in the procedural complications between the two groups [38].

Either RF or cryoballoon can be used for LAAI. When using RF ablation, LAAI can be achieved by wide-area linear ablation (including left atrial anterior line, left atrial roof line and left atrial mitral isthmus line). When using cryoballoon, similarly to PVI, LAAI can be achieved by directly occluding and freezing the LAA ostia. Recent non-randomized study found that LAAI can be more readily achieved by using cryoballoon, whereas the 1-year ATa recurrence-free rate was significantly higher in patients with LAAI following RF-guided wide area LAAI (RF:76.3% vs. CB:56.7%, *p* = 0.0017) [39].

Evidence has shown that electrical LAAI can be associated with increased risk of LAA thrombosis even under sinus rhythm because of loss of the LAA contractibility [40,41]. Recent studies from our group demonstrated that among patients after LAAI, a strategy of left atrial appendage occlusion (LAAO) was correlated with significantly less thromboembolic events as compared with oral anticoagulant (OAC) [42].

The limited efficacy of endocardial CA for persistent and long-standing persistent AF led to the development of a minimally invasive hybrid convergent (epicardial/endocardial) ablation approach in order to achieve more comprehensive, durable, transmural lesions. The recent CONVERGE trial randomized 153 patients with persistent and long-standing persistent AF to undergo Hybrid Convergent ablation or endocardial CA [43]. During the 1-year follow-up, the rate of freedom from AF/AT was significantly higher in the Hybrid Convergent group than that in the endocardial CA group (67.7% vs. 50.0%) [43]. At 1.5 years assessed by 7-day Holter, 74% in the Hybrid Convergent group and 55% in the endocardial CA patients experienced ≥90% AF burden reduction [43]. However, patients in the Hybrid Convergent group had more major adverse events (2.9% within one week post-procedure, and 4.9% between one week and 1month post-procedure) compared to endocardial CA group (0%) [43].

For patients with persistent AF, PVI remains the foundation ablation strategy. As above discussed, there have been studies showing improved outcome when PVI combined with different ablation strategies in selected patient group; besides, other ablation concepts, such as rotor ablation, and cardioneuroablation, have also been proposed. The additional merit of such ablation strategies in persistent AF still needs to be warranted. Questions with respect to proper patient selection, optimal timing to perform adjunctive ablation, choosing of pertinent ablation strategy, and selection of efficacious and safe ablation technology, remain to be answered (Figure 4).

## 5. Effect of CA on Hard Clinical Endpoints in AF

CA has been shown to improve quality of life (QoL) in AF patient [44]. Several recent randomized trials have investigated the effects of CA on clinical hard endpoints. The CASTLE-AF trial randomized AF patients with heart failure (HF) to receive either CA or medical therapy [45]. The patients mainly had persistent or LS persistent AF, and all patients had New York Heart Association (NYHA) class II-IV heart failure, LVEF ≤ 35%, and implanted defibrillator. During a median follow-up of 37.8 months, patients in the CA group had significantly lower all-cause mortality (13.4% vs. 25.0%), less HF hospitalization (20.7% vs. 35.9%), and lower cardiovascular mortality (11.2% vs. 22.3%). Notably, the subgroup analyses showed that patients with relatively younger age (<65%), less severe HF (NYHA II and LVEF ≥ 25%) benefited significantly from CA [45].

The recent large CABANA trial randomized 2204 AF patients to undergo CA or receive medical therapy [46]. Notably, 27.5% of the patients in the medication group ultimately crossed over and received CA. During a median follow-up of 48.5 months, the results per intention-to-treat (ITT) analysis showed similar all-cause mortality (5.2% vs. 6.1%), less combined death and cardiovascular hospitalization (51.7% vs. 58.1%), and higher freedom from AF recurrence (50.1% vs. 30.5%). However, the subgroup analysis focusing on younger (<65 years) patients demonstrated significantly less primary endpoint events associated with CA as relative to medical therapy [46].

Moreover, in the CABANA Heart Failure subgroup analysis, where patients with mainly persistent AF, NYHA II, and LVEF > 35% were included, CA group had a significant (36%) reduction in the primary endpoint and a significant (43%) reduction in all-cause mortality compared to medical therapy during a median follow-up of 48.5 months [47]. 

The recent CAPA trial randomized 648 patients with persistent and long-standing persistent AF (mean age 65 years, mean LAD 46 mm, mean LVEF 53%) to CA or pharmacotherapy [48]. During a mean follow-up of 54.2 months, patents in the CA group had significantly lower rate of stroke (4.2% vs. 7.2%), lower new-onset HF (2.8% vs. 7.2%), and higher freedom from ATa recurrence (60.6% vs. 20.9%) [48].

**Figure 5:**
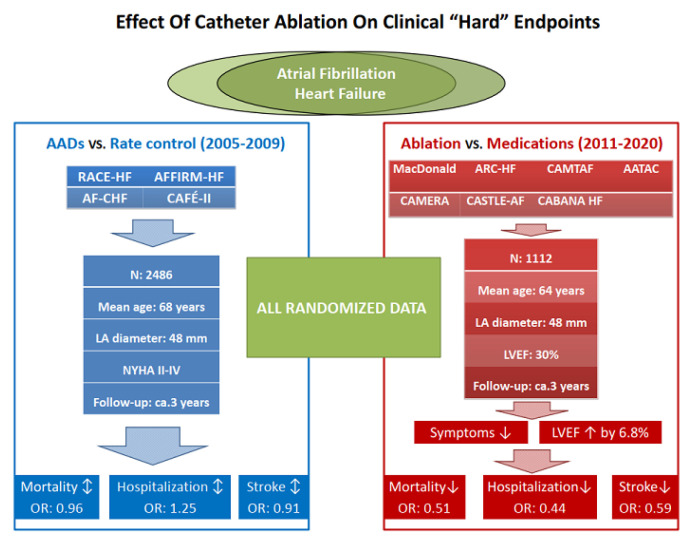
Effect Of Catheter Ablation on Clinical “Hard” Endpoints.

A recent meta-analysis included seven RCTs involving 1112 AF patients with HF (mean age 64 years, mean LVEF 30%, mean LAD 48 mm) [49]. The pooled-analysis demonstrated that CA as a rhythm control strategy substantially improved overall survival rate, reduced rehospitalization, increased the freedom from ATa recurrence, preserved cardiac function, and improved QoL during a mean of 2.7 years follow-up [49] (Figure 5).

## 6. CA and AF Burden (Figure 6)

Historically, ATa recurrence (or AF ablation failure) was arbitrarily defined as EG documentation of any episode of ATa ≥ 30 s. However, only brief AF episode may not be linked to clinical events [50]. The concept of AF burden refers to the amount of AF a patient has. That is, AF is regarded as a quantitative entity and not merely as a binary condition (presence or absence of AF). The definition for AF burden is the proportion of time an individual in AF during a period of continuous ECG monitoring [51]. Large scale study has demonstrated that greater burden of AF is associated with a higher risk of ischemic stroke independent of known stroke risk factors [52].

**Figure 6:**
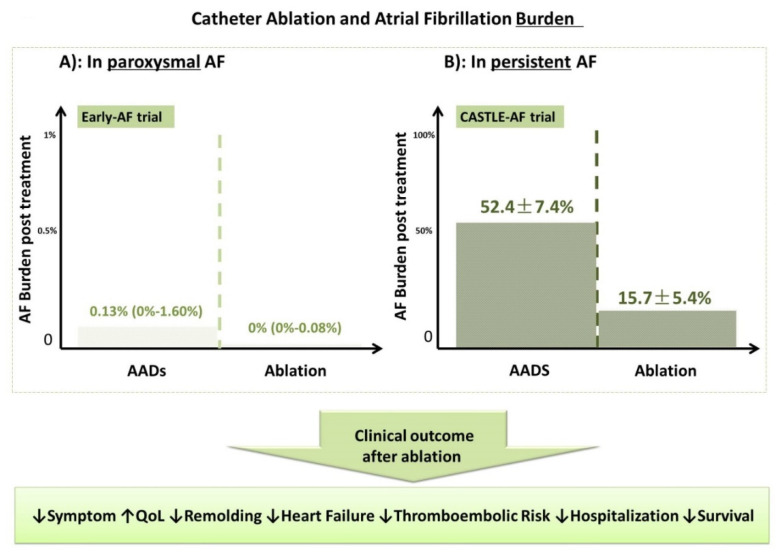
Catheter Ablation and Atrial Fibrillation Burden.

### 6.1. CA and AF Burden in Paroxysmal AF

In the early AF trial [7], 303 treatment naïve patients with paroxysmal AF were randomized to undergo initial CA or optimal AADs therapy; all patients were implanted with an implantable cardiac monitor (ICM). One year follow-up showed that the mean AF burden (the percentage of total time in AF) was 0.6 ± 3.3% in patients in CA group and 3.9 ± 12.4% in those in AADs group [7] (Figure 6).

The CIRCA-DOSE trial randomized 346 patients with drug-refractory paroxysmal AF to different ablation technologies: contact force-guided RF (CF-RF; n = 115), 4 min CB (Cryo-4; n = 115), or 2 min CB (Cryo-2; n = 116) ablation [14]. All patients were implanted with ICM minimum of 30 days before the index ablation procedure. Compared with the monitoring before ablation, AF burden was reduced by a median of 99.3% (interquartile range, 67.8–100.0%) in the CF-RF group, 99.9% (interquartile range, 65.3–100.0%) in the CB-4 group, and 98.4% (interquartile range, 56.2–100.0%) in the CB-2 group during the one-year follow-up [14].

The CLOSE to CURE study was a prospective single armed study [53]. A total of 105 patients with paroxysmal AF after failed AADs therapy were implanted with ICM two months before CA. Ablation was performed using contact force RF catheter following CLOSE protocol. The median ATa burden was decreased from 2.68% (0.09–15.02%) at baseline to 0% (0–0%) during the first year and to 0% (0–0%) during the second year, with significant reduction in ATa burden by 100% [100–100%] [53].

### 6.2. CA and AF Burden in Persistent AF

The CASTLE-AF trial randomized AF (mainly Per-AF) patients with HF (mean LVEF 32%) to CA (n = 179 patients) or medical therapy (n = 184 patients). All patients were implanted with implantable cardioverter-defibrillator (ICD) or cardiac resynchronization therapy plus defibrillator CRT-D. Sixty months follow-up showed significant reduction of AF burden from 62% (0–99%) to 0% (0–62%) in CA group, whereas significant increased AF burden from 64% (0–99%) to 99% (4–99%) was observed in the medical therapy group [45]. Post hoc analysis demonstrated that the AF burden, but not recurrent ATa > 30 s after CA, was a strong predictor for primary composite endpoint and all-cause mortality [54] (Figure 6).

The AMICA Trial randomized patients with persistent (or longstanding) AF and LVEF ≤ 35% to CA or medical therapy [55]. Seventy-five patients had either ICD or CRT-D device. Device-recorded AF burden was 0% or maximally 5% of the time in 72% of the patients in CA group vs. 44% AF burden among the patients in the medical therapy group during the one year follow-up [55].

## 7. Ablation Technologies and Concepts (Figure 7)

New ablation concepts or technologies are continuously under development for efficacious lesion formation, safety, and facilitated procedure.

**Figure 7:**
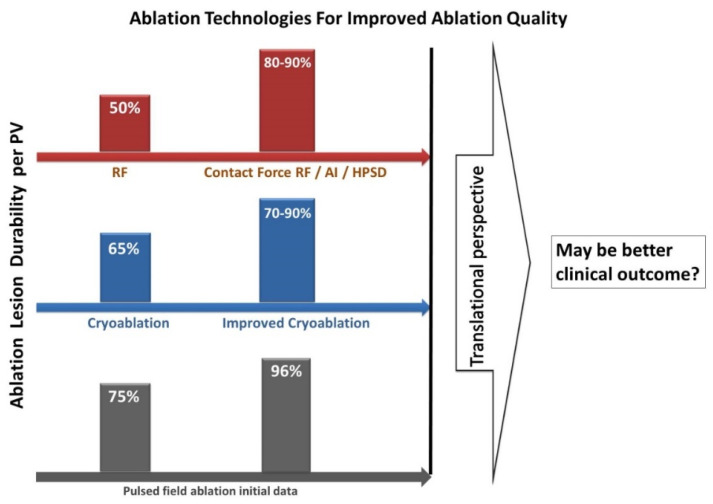
Ablation Concepts Additionally, Technologies For Improved Ablation Quality.

Cryoballoon (CB) as a single shot device has been demonstrated as effective as traditional RF ablation for PVI. Nowadays, CB has established as a simplified index approach in treating AF. The repeat procedure data from the previous FIRE AND ICE Trial revealed that 30–60% of the treated PVs using either CB1/CB2 or non-contact force RF catheters were reconnected during a mean of 6 months follow-up [31]. Remap data from our group after time to isolation (TTI) guided CB2 PVI at one year, showing 12.5–30.8% PV reconnection dependent on the index freeze duration, and target freeze durations of 4 min. vs. 3 min. were associated with significantly increased lesion durability (87.5% vs. 69.2%, per PV), without compromising procedure safety [56,57].

Recently, the so called ultra-low temperature cryoablation has been developed and allows operators to ablate tissue using ultra-low temperatures with a theoretical minimum of −196 °C [57]. Preclinical study demonstrated single-shot electrogram elimination rate >90% in atrium and in ventricle, and lesion depth can be achieved at 2 mm in atrium and 5.6 mm in ventricular [57]. Re-map and histology study at 3 months demonstrated transmural and durable ablation lesions [58]. Nonetheless, these promising results still need to be warranted.

A contiguous, transmural lesion set by ablation without collateral injury to adjacent tissues is crucial for successful PVI. Conventional RF ablation used low-to-moderate power (25–35 W), long-duration settings (30–60 s per site) to achieve effective lesions by conductive heating. A different ablation strategy, the so-called high-power short-duration (HPSD) RF ablation (usually: 45–50 W, 2–10 s per site at the posterior wall and 5–15 s per site at the anterior wall) have gained great interest and showed improved ablation efficiency and low complications based on the shifting of energy balance from conductive heating to resistive heating. Recent randomized or comparative studies showed that using HPSD ablation conferred significantly shorter RF and procedural time and led to a good ablation outcome [59,60,61].

Our initial experience employing ablation index (AI) guided 50 W PVI (targeting AI: 550/400 anterior/posterior, inter-lesion distance: 6 mm) showed a very high rate (>90%) of first-pass isolation, remarkably short RF time (11 min, and RF time at posterior wall only 3 min), short procedural time, and good clinical outcome (at 15 months: 89.4% freedom from recurrence among paroxysmal AF patients, and 80.4% freedom from recurrence among persistent AF patients); importantly, such controlled HPSD PVI approach seemed to be associated with lower rate of esophageal complication (3% mild lesion) as compared to those with conventional power [62,63,64,65,66]. Recent data also showed that HPSD ablation (45–50 W, 15 s/8 s at anterior/posterior) resulted in significantly improved durable PVI as compared to conventional power ablation (83.4% vs. 47.8%, at 1.2 years follow-up) [67].

Pulsed field ablation (PFA) is a novel nonthermal ablation technology to treat AF. PFA has gained great interest given its notable safety and efficacy profile, i.e., myocardial tissue selectivity and unique ability to reduce the risk of collateral tissue damage. Preclinical experiments have demonstrated that PFA based PVI and SVC isolation were safe and efficacious, without injuring adjacent nerves, vessel tissues, or the esophagus [68,69]. The initial clinical trials (IMPULSE and PEFCAT) showed a one-year single procedure clinical success rate of 87% after PFA PVI in paroxysmal AF [70]. The recent PersAFOne trial further assessed the safety and lesion durability of PFA for both PVI and left atrial posterior wall isolation in persistent AF, and the initial results showed 96% durable PVI and 100% durable PWI at 3 months remapping procedure after optimized PFA [71] (Figure 7). Besides, these initial clinical trials also showed very promising safety profile of PFA, typical thermal ablation energy related complications, such as esophageal injury, phrenic nerve palsy, or PV stenosis were not observed [70,71,72,73]. Currently, this new technology remains at an early stage, and basic and clinical researches are still needed to further improve the system design and define the optimal ablation dosing at different cardiac region.

## 8. Safety Considerations

Safety is one major consideration with respect to catheter ablation. Potential adverse events related to ablation can be: death, atrio-esophageal fistula, cardiac tamponade, thromboembolic events, bleeding, worsening heart failure, pulmonary vein stenosis, phrenic nerve palsy, bradycardia, hypotension, ventricular tachyarrhythmia, or vascular access complications, etc.

Recently pooled analysis from our group found a low and similar rate of overall serious adverse events related to catheter ablation vs. AADs (5.6% vs. 4.9%) [12]. The most serious adverse event in the ablation group was cardiac tamponade (1.4%), whereas the most serious adverse event in the medication group was bradycardia (1.4%), followed by syncope (1%) and worsening heart failure (0.6%) [12].

Recent large sample, real-world registry studies revealed the incidence of cardiac tamponade around 1% [74,75]. Notably, RF or RF-based excessive ablation seemed to be associated with significantly higher risk of cardiac tamponade as compared to balloon-based ablation modalities, in addition, operators’ inexperience, or patients’ characteristics, e.g., female gender, advanced age, or multiple chronic comorbidities, were identified as independent risk factors for cardiac tamponade [74,75]. Early identification, successful pericardiocentesis, streamlined protocol, and cardiac surgery backup are the keys for good management of cardiac tamponade.

Cryoballoon-based PVI has been established as an effective treatment for AF, and the most frequent adverse event during cryoballoon-based PVI is phrenic nerve palsy (PNP). The recent large multicenter YETI registry enrolling more than 17,000 patients’ data revealed that the incidence of PNP during cryoballoon-based PVI was 4.2%, of them more than 50% of the patients had recovered PN function at the end of the procedure, and 97% of the patients had recovered PN function at 12 months. Notably, only around 2.3% of the involved patients reported symptomatic PNP and 0.06% of them showed symptomatic permanent PNP [76]. These findings seemed to be in line with the single center observation from our group [77]. Currently the practical approach to monitor phrenic nerve function is direct phrenic stimulation/capture and observing the diaphragmatic motion during ablation [78].

Atrio-esophageal fistula (AEF) represents a seldom but dramatic complication related by catheter ablation of AF particularly when treating the left atrium posterior wall. A recent French nationwide survey reported a rate of 0.026% (33/129, 286) of AEF after AF/AT ablation, stating that all cases of AEF occurred after RF ablation, and the incidence of AEF retained despite the introduction of RF contact force catheter or esophageal temperature monitor [79]. The study further revealed that repeated CT scan may increase the sensitivity of the diagnosis of AEF, and the mortality after AEF may be decreased to 31% if treated early with corrective surgery, otherwise the mortality can reach a high of up to 93% if only treated with medications or stenting [79]. Anatomic proximity between left atrium and esophagus, and “over-shooting” during energy delivery at the posterior wall are important risk factors for esophageal thermal lesions after ablation. Experience from our group has suggested that a novel tailored high-power (50 W) short-duration ablation strategy guided by ablation index may lead to a very fast and effective PVI, with very short RF time, particularly at the posterior wall, and remarkably low incidence of esophageal thermal lesion [62,63,64,65].

Cryoballon-based AF ablation can also cause AEF. John et al. investigated the incidence of AEF after cryoballon based AF ablation; eleven AEF out of more than 120,000 cases (0.009%) were identified, with mortality of 64% among the patients with AEF [80]. Notably in this study all cases of AEF occurred in relation to the treatment of left PVs, suggesting that the proximity of the esophagus to the left atrium and esophageal luminal temperature cooling particularly when freezing the left PVs merit attention [79]. Nonetheless, AEF after AF ablation is still extremely rare, difficult to be predicted, but fatal. Development of novel ablation energy sources, e.g., PFA, may further offer promising perspectives to avoid those thermal energy related complications.

## 9. Other Aspect: Gender Differences

Existing data have shown gender differences in utilization of catheter ablation for AF. As compared to male patients, female patients seemed to be referred late and less frequently for catheter ablation, and the age of female patients referred for ablation appears to be older and have lower body-mass-index, smaller left atrial dimension, but larger LAD indexed by the body-surface-area [81]. In a US nationwide analysis, female patients were 17% less likely to receive AF ablation procedures [82]. Notably in the previously published randomized trials, the enrollment of female patients seemed to be under-representative. A pooled-analysis of AF ablation clinical trials found that female patients account for about one-fifth of the study population [83].

There are also gender differences in complications following catheter ablation of AF. A recent analysis using national data from Australia and New Zealand found that females undergoing AF ablations experienced a 25% higher risk of procedural complications compared with males, which was mainly driven by increased risk of vascular injury, pericardial effusion, and bleeding [84].

Recent study also showed that female gender seemed to be significantly associated with arrhythmia recurrence after multiple ablation procedures for persistent AF despite that PV reconnection was less likely and fewer reconnected PVs occurred in female patients. Further studies are needed to better understand the mechanisms responsible for AF recurrence and close the knowledge gaps in terms of gender difference [85].

## 10. Summary (Figure 8 Graphic Summary)

**Figure 8:**
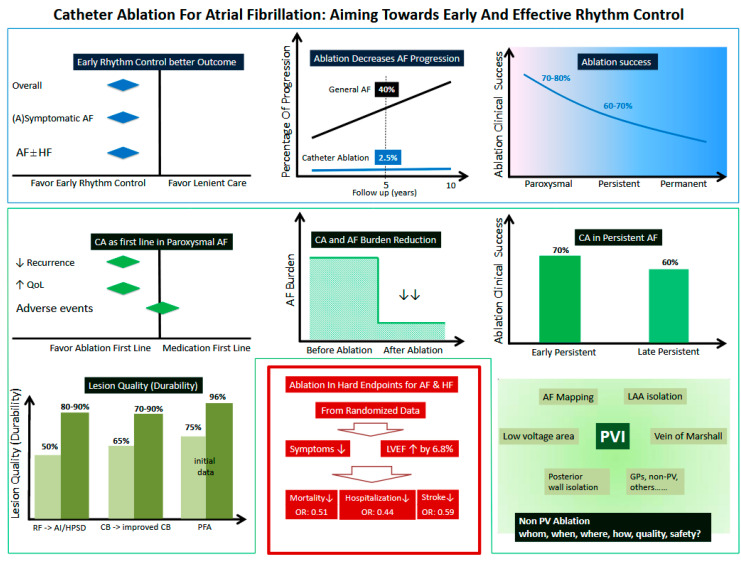
Graphic Summary Chen et al.

Catheter Ablation (CA) is an effective therapeutic option in treating atrial fibrillation (AF). Importantly, recent data show that CA as a rhythm control strategy not only significantly reduces the AF burden, but also substantially improves clinical hard endpoints. Since AF is a progressive disease, the time of Diagnosis-to-Intervention appears crucial. Recent evidence shows that earlier rhythm control is associated with a lower risk of adverse cardiovascular outcomes in patients with early AF. Particularly, CA as an initial first line rhythm control strategy is associated with significant reduction of arrhythmia recurrence and re-hospitalization in patients with paroxysmal AF. CA is shown to significantly lower the risk of progression from paroxysmal AF to persistent AF. When treating persistent AF, the overall clinical success after ablation remains unsatisfactory, however the ablation outcome in patients with “early” persistent AF appears better than those with “late” persistent AF. “Adjunctive” ablation on top of pulmonary vein isolation (PVI), e.g., ablation of atrial low voltage area, left atrial posterior wall, vein of Marshall, left atrial appendage, etc. may further reduce arrhythmia recurrence in a selected patient group. New ablation concepts or new ablation technologies have been developing to optimize therapeutic effects or a safety profile, and may ultimately improve the clinical outcome (Figure 8).

## Data Availability

Not applicable.
